# Evaluation of Social Acknowledgment and Mental Health Among Kurdish Survivors of Genocide in 1988

**DOI:** 10.1001/jamanetworkopen.2023.28793

**Published:** 2023-08-14

**Authors:** Sina Neldner, Razaw Noori, Harem Nareeman Mahmood, Frank Neuner, Hawkar Ibrahim

**Affiliations:** 1 Clinical Psychology and Psychotherapy Unit, Department of Psychology, Bielefeld University, Bielefeld, Germany; 2 Independent Researcher, Halabja, Kurdistan Region, Iraq; 3 Department of Clinical Psychology, Koya University, Koya, Kurdistan Region, Iraq; 4 Vivo International, Konstanz, Germany

## Abstract

This cross-sectional study assesses whether higher levels of trauma were associated with less perceived social acknowledgment and higher psychopathology among Kurdish survivors of a chemical attack that took place in 1988.

## Introduction

Previous research has extensively investigated the psychosocial consequences of several genocidal events, such as the Holocaust and the genocides in Sudan and Rwanda.^1,2,3^ However, the psychological aftermath of the Kurdish genocide, specifically the chemical attack against the city of Halabja in the Kurdistan Region of Iraq in 1988, remains relatively understudied. This study focuses on the psychosocial situation of Kurdish survivors after over 30 years. Given that survivors previously reported stigmatization as a result of their experiences,^4,5^ we anticipated that more genocide-related trauma would be associated with less perceived social acknowledgment and higher psychopathology.

## Methods

Using snowball sampling, structured interviews in this cross-sectional study were conducted with 143 survivors of the chemical attack. Oral informed consent was obtained after reading a standardized document containing study information. Valid and reliable Kurdish versions of the Posttraumatic Stress Disorder (PTSD) Checklist for *DSM-5* (PCL-5) and the depression section of the Hopkins Symptom Checklist (D-HSCL-25)^6^ were used to evaluate symptoms of PTSD and depression. Potential traumatic events that occurred before, during, and after the chemical attack were assessed with the War and Adversity Exposure Checklist (eTable 1 in Supplement 1). Furthermore, the Social Acknowledgment Scale was developed to measure the level of acknowledgment survivors perceived by their family and community (eTable 2 in Supplement 1). Ethical and institutional board approval were granted by Bielefeld University (Germany) and Koya University (Iraq). The study followed the STROBE reporting guideline. Mediation analysis was performed with SPSS, version 29.0 (IBM Corp) from June 28 to July 2, 2023. All tests were 2-sided, and *P* < .05 was considered statistically significant.

## Results

The 143 participating survivors reported having experienced a mean (SD) of 5.07 (3.05) traumatic event types (range, 1-16) related to the genocide and a mean (SD) of 10.34 (4.06) over their entire life span (range, 2-24); 133 survivors (93.0%) rated the chemical attack as the worst event they had ever experienced. Approximately half of the survivors met the probable diagnostic criteria for PTSD (51.7% [74 of 143]) and depression (49.0% [70 of 143]), when the cutoff scores of the Kurdish versions of PCL-5 (>23.0) and D-HSCL (>1.75) were applied (Table 1). In mediation analysis, the association between genocide-related trauma and psychopathology was found to be partially mediated by perceived social acknowledgment, also after adjusting for potential confounding factors (ie, gender and education) (Table 2).

**Table 1.** Sociodemographic Information, Trauma Exposure, and Psychopathology

Characteristic	No. (%) (N = 143)
Gender	
Female	86 (60.1)
Male	57 (39.9)
Age, mean (SD) [range], y	51.4 (12.5) [36.0-92.0]
<40	18 (12.6)
40-49	58 (40.5)
50-59	32 (22.4)
>59	35 (24.5)
Age during chemical attack, mean (SD) [range], y	20.0 (12.49) [5-61]
<10	32 (22.4)
10-19	59 (41.2)
20-39	38 (26.6)
>39	14 (9.8)
Formal education, mean (SD) [range], y	6.8 (6.2) [0-20.0]
0	44 (30.8)
1-5	25 (17.5)
6-10	28 (19.5)
>10	46 (32.2)
Work status	
At home	68 (47.6)
Full time	31 (21.7)
Part time	36 (25.1)
Retired	8 (5.6)
Regular Income	
Yes	84 (58.7)
No	59 (41.3)
Marital status	
Never married	7 (4.9)
Currently married	118 (82.5)
Divorced or separated	2 (1.4)
Widowed	16 (11.2)
Children, mean (SD) [range], No.	4.0 (2.3) [0-10.0]
0-2	36 (25.2)
3-5	73 (51.0)
>5	34 (23.8)
Close family members directly affected by chemical attack, mean (SD) [range], No.	6.3 (3.8) [0-33.0]
0-3	26 (18.2)
4-9	97 (67.8)
>9	20 (14.0)
Lifetime displacements, mean (SD) [range], No.	1.7 (1.1) [0-6.0]
0-1	76 (53.1)
2-3	58 (40.6)
>3	9 (6.3)
Traumatic experiences, mean (SD) [range], No.	
Before genocide	3.5 (3.0) [0-15.0]
During genocide	5.1 (3.1) [1.0-15.0]
After genocide	6.54 (3.75) [0-16]
PTSD symptom level, mean (SD) [range]^a^	28.7 (18.8) [0-71.0]
Depression symptom level, mean (SD) [range]^b^	1.8 (0.7) [1.0-3.6]

Abbreviation: PTSD, posttraumatic stress disorder.

^a^ Total score range for Posttraumatic Stress Disorder Checklist for *DSM-5*, 0 to 80.0. Higher scores indicate higher symptom levels.

^b^ Mean score range for depression section of the Hopkins Symptom Checklist, 1.0 to 4.0. Higher scores indicate higher symptom levels.

**Table 2.** Mediation Analysis: Role of Social Acknowledgment in the Association Between Genocide-Related Trauma and Mental Health Symptomatology^a^

Outcome	Standardized regression coefficient β		
	Total effect (pathway c)	Direct effect (pathway c’)	Indirect effect (pathway ab)
Crude analysis			
PTSD symptoms	0.28^b^	0.21^c^	0.07 (0.02-0.14)^d^
Depression symptoms	0.23^b^	0.16^e^	0.06 (0.01-0.13)^d^
Adjusted analysis			
PTSD symptoms	0.32^b^	0.25^c^	0.07 (0.02-0.13)^d^
Depression symptoms	0.26^b^	0.20^e^	0.06 (0.01-0.12)^d^

Abbreviation: PTSD, posttraumatic stress disorder.

^a^ Mediation analysis was performed for 133 survivors. The crude analysis represents unadjusted data, while the adjusted analysis incorporates gender and education as covariates to account for their potential effects. For pathway a, the association of genocide-related trauma load with mediator (social acknowledgment) was significant (crude: β = −0.24; adjusted: β = −0.27 [*P* < .001]). For pathway b, associations of the mediator with outcomes were significant for PTSD (crude: β = −0.30; adjusted: β = −0.25 [*P* < .01]) and depression symptoms (crude: β = −0.26; adjusted: β = −0.21 [*P* < .01]).

^b^ *P* < .001.

^c^ *P* < .01.

^d^ The 95% CIs are based on 5000 bootstrap resamples.

^e^ *P* < .05.

## Discussion

In this study investigating long-term consequences of the chemical attack, survivors had high levels of trauma exposure and psychopathology. Findings underscore the integral role of social acknowledgment in their psychosocial well-being. Survivors with high levels of genocide-related trauma perceived less acknowledgment and demonstrated elevated levels of psychopathology.

The absence of social acknowledgment may originate from societal stigma and misconceptions surrounding traumatic events and their consequences. Survivors often face rejection and stigmatization when articulating their experiences and emotional responses to the genocide,^5^ which may amplify psychological distress and hinder recovery.

Support initiatives should thus prioritize enhancement of social acknowledgment within familial and community settings. Education programs aimed at dispelling misconceptions about the psychological and physical consequences of genocide can help reduce stigma and promote compassion toward survivors. Community-based interventions, such as support groups and commemorative events, can further provide secure environments for survivors to share their narratives, promote unity, and facilitate healing.

Study limitations include the lack of sample representativeness, the cross-sectional design preventing causal inferences, and the incompleteness regarding other context variables (eg, economic resources). Further research is needed to delve deeper into the complex dynamics of social acknowledgment and its impact on survivors’ mental health over extended periods. Longitudinal studies and interventions integrating individual, family, and community-based approaches can provide valuable insights into the development of comprehensive support systems for survivors and their families. Addressing social acknowledgment could significantly contribute to enhancing the psychosocial well-being and resilience of genocide survivors.

## Notes

Supplement 1. **eTable 1.** War and Adversities Exposure Checklist (English Version) **eTable 2.** Social Acknowledgment Scale
eReferences.

Supplement 2.
Data Sharing Statement

## References

[1] Barel E, Van IJzendoorn MH, Sagi-Schwartz A, Bakermans-Kranenburg MJ. Surviving the Holocaust: a meta-analysis of the long-term sequelae of a genocide. Psychol Bull. 2010;136(5):677-698. doi:10.1037/a0020339 20804233

[2] Meffert SM, Marmar CR. Darfur refugees in Cairo: mental health and interpersonal conflict in the aftermath of genocide. J Interpers Violence. 2009;24(11):1835-1848. doi:10.1177/0886260508325491 18945917 PMC6777844

[3] Schaal S, Weierstall R, Dusingizemungu JP, Elbert T. Mental health 15 years after the killings in Rwanda: imprisoned perpetrators of the genocide against the Tutsi versus a community sample of survivors. J Trauma Stress. 2012;25(4):446-453. doi:10.1002/jts.21728 22865747

[4] Ibrahim H, Ertl V, Catani C, Ismail AA, Neuner F. Trauma and perceived social rejection among Yazidi women and girls who survived enslavement and genocide. BMC Med. 2018;16(1):154. doi:10.1186/s12916-018-1140-5 30208905 PMC6136186

[5] Moradi F, Moradi F, Söderberg M, Olin AC, Lärstad M. Gendered lived experiences of marriage and family following exposure to chemical warfare agents: content analysis of qualitative interviews with survivors in Halabja, Kurdistan-Iraq. BMJ Open. 2020;10(10):e034277. doi:10.1136/bmjopen-2019-034277 33055109 PMC7559057

[6] Ibrahim H, Ertl V, Catani C, Ismail AA, Neuner F. The validity of Posttraumatic Stress Disorder Checklist for DSM-5 (PCL-5) as screening instrument with Kurdish and Arab displaced populations living in the Kurdistan region of Iraq. BMC Psychiatry. 2018;18(1):259. doi:10.1186/s12888-018-1839-z 30115040 PMC6097219

