# Supplementary material for: Evaluation of Social Acknowledgment and Mental Health Among Kurdish Survivors of Genocide in 1988
Source: JAMA Netw Open. 2023 Aug 14;6(8):e2328793. doi: 10.1001/jamanetworkopen.2023.28793 (PMC10425822; doi:10.1001/jamanetworkopen.2023.28793)
Supplement: Supplement 1. — eTable 1. War and Adversities Exposure Checklist (English Version) eTable 2. Social Acknowledgment Scale eReferences. [file jamanetwopen-e2328793-s001.pdf]

Supplemental Online Content

Neldner S, Noori R, Mahmood HN, Neuner F, Ibrahim H. Evaluation of social acknowledgment and mental health among Kurdish survivors of genocide in 1988. *JAMA Netw Open*. 2023;6(8):e2328793. doi:10.1001/jamanetworkopen.2023.28793

eTable 1. War and Adversities Exposure Checklist (English Version)
eTable 2. Social Acknowledgment Scale
eReferences

This supplemental material has been provided by the authors to give readers additional information about their work.

© 2023 Neldner S et al. *JAMA Network Open*.

eTable 1. War and Adversities Exposure Checklist[1] (English Version)

People who come from / live in countries affected by conflict, war and persecution often have experienced stressful and terrifying events. I would like to ask you about such events. I will read several experiences to you. Please indicate, whether you have experienced or witnessed the respective event or not.

#	Events	Yes	No
1	Have you ever been severely deprived of food and water due to war or flight?	1	0
2	Have you ever been exposed to armed combat (for example, fighting with guns or artillery, shelling)?	1	0
3	Have you ever witnessed an execution?	1	0
4	Have you ever seen a dead body (apart from funerals) or a rotting corps?	1	0
5	Have you ever witnessed someone being killed?	1	0
6	Have you ever been imprisoned or kidnapped?	1	0
7	Have you ever witnessed a bombing, burning, or violent destruction of residential areas?	1	0
8	Have you ever been forced to separate from first grade family members because of the war?	1	0
9	Have you ever lost (death or disappearance) anyone close to you because of the war?	1	0
10	Have you ever been tortured?	1	0
11	Have you ever experienced a natural disaster (for example, flood, hurricane, tornado, earthquake) that was life threatening to you or others?	1	0
12	Have you ever experienced a fire or explosion that was life threatening to you or others?	1	0
13	Have you ever experienced a transportation accident (for example, a car accident, boat accident, train, plane crash) that was life threatening to you or others?	1	0
14	Have you ever witnessed such a severe transportation accident?	1	0
15	Have you ever been physically assaulted (for example, being attacked, hit, slapped, kicked, beaten up) by someone outside your family?	1	0
16	Have you ever witnessed someone being assaulted physically in this way?	1	0
17	Have you ever been physically assaulted in this way by a family member?	1	0
18	Have you ever been assaulted with a weapon (for example, being shot, stabbed, threatened with a knife, gun, bomb)?	1	0
19	Have you ever witnessed someone being assaulted with a weapon (for example, being shot, stabbed, threatened with a knife, gun, bomb)?	1	0
20	Have you ever been sexually assaulted (for example, rape, attempted rape, made to perform any type of sexual act through force or threat of harm) by someone outside your family?	1	0
21	Have you ever witnessed such a sexual assault?	1	0
22	Have you ever been sexually assaulted in this way by your husband or another family member?	1	0
23	Have you made any other unwanted sexual experience?	1	0
24	Have you ever witnessed someone making an unwanted sexual experience?	1	0
25	Have you ever experienced a life-threatening illness or injury?	1	0
26	Have you ever seen someone who suffered from a life-threatening illness or injury?	1	0

© 2023 Neldner S et al. *JAMA Network Open.*

eTable 2. Social Acknowledgment Scale

Item No.	Items	Factor
1	My family showed empathy for what I went through.	(1) Family
2	My family can understand what happened to me.	(1) Family
3	My family is grateful that I have survived.	(1) Family
4	When I express my emotions on what happened, people are blaming me.	(2) Community
5	When I talk about what happened to me, most people think I am exaggerating.	(2) Community

Note: The scale was developed based on previous literature (Maercker & Müller, 2004),[2] qualitative interviews with survivors and expert panels with local psychologists. Items and factor structure are based on the results of Explorative Factor Analysis. Internal consistencies for the overall questionnaire: McDonald's ω = .66; 95% CI [.50, .76]; Factor 1: ω = .80; 95% CI [.74, .86], Factor 2: ω = .74; 95% CI [.62, .83].

eReferences

1. Ibrahim H, Ertl V, Catani C, Ismail AA, Neuner F. Trauma and perceived social rejection among Yazidi women and girls who survived enslavement and genocide. *BMC Med*. 2018;16(1):154. doi:10.1186/s12916-018-1140-5
2. Maercker A, Müller J. Social acknowledgment as a victim or survivor: A scale to measure a recovery factor of PTSD. *J Trauma Stress*. 2004;17(4):345-351. doi:10.1023/B:JOTS.0000038484.15488.3d

© 2023 Neldner S et al. *JAMA Network Open*.
