# Supplementary material for: Evaluation of Social Acknowledgment and Mental Health Among Kurdish Survivors of Genocide in 1988
Source: JAMA Netw Open. 2023 Aug 14;6(8):e2328793. doi: 10.1001/jamanetworkopen.2023.28793 (PMC10425822; doi:10.1001/jamanetworkopen.2023.28793)
Supplement: Supplement 2. — Data Sharing Statement [file jamanetwopen-e2328793-s002.pdf]

Data Sharing Statement

Neldner. Evaluation of Social Acknowledgment and Mental Health Among Kurdish Survivors of Genocide in 1988. *JAMA Netw Open*. Published August 14, 2023.
doi:10.1001/jamanetworkopen.2023.28793

Data
Data available: No

Additional Information
Explanation for why data not available: The datasets generated and/or analyzed during the study are not publicly available due to terms of consent to which the participants agreed but are available from the corresponding author upon reasonable request.
